# Complementary insights from wastewater surveillance and the toxicology findings of clinical presentations involving new psychoactive substances in Australia

**DOI:** 10.1093/jat/bkag071

**Published:** 2026-08-05

**Authors:** Richard Bade, Dhayaalini Nadarajan, Keith Harris, Christine Harvey, Thanjira Jiranantakan, Hannah Morgan, Emma Partridge, Jennifer Schumann, Jennifer L Smith, Courtney Weber, Wayne Hall

**Affiliations:** Queensland Alliance for Environmental Health Sciences (QAEHS), The University of Queensland, Dutton Park, Brisbane, Queensland, 4102, Australia; Queensland Alliance for Environmental Health Sciences (QAEHS), The University of Queensland, Dutton Park, Brisbane, Queensland, 4102, Australia; Clinical Toxicology Unit, Princess Alexandra Hospital, Brisbane, Queensland, 4102, Australia; Faculty of Medicine, University of Queensland, Brisbane, Queensland, 4106, Australia; Centre for Alcohol and Other Drugs, New South Wales Ministry of Health, Sydney, New South Wales, 2065, Australia; Centre for Alcohol and Other Drugs, New South Wales Ministry of Health, Sydney, New South Wales, 2065, Australia; New South Wales Poisons Information Centre, Sydney Children’s Hospitals Network, Sydney, New South Wales, 2145, Australia; Faculty of Medicine and Health, University of Sydney, Sydney, New South Wales, 2050, Australia; Edith Collins Centre, Drug Health Services, Royal Prince Alfred Hospital, Sydney, New South Wales, 2050, Australia; Centre for Alcohol and Other Drugs, New South Wales Ministry of Health, Sydney, New South Wales, 2065, Australia; Forensic Science SA (Toxicology), Adelaide, South Australia, 5000, Australia; College of Science and Engineering, Flinders University, Bedford Park, South Australia, 5042, Australia; Department of Forensic Medicine, Monash University, Melbourne, 3006, Australia; Victorian Institute of Forensic Medicine, Melbourne, 3006, Australia; Monash Addiction Research Centre, Eastern Clinical School, Frankston, 3199, Australia; Medical School, The University of Western Australia, Perth, WA, 6009, Australia; Centre for Clinical Research in Emergency Medicine, Harry Perkins Institute of Medical Research, Perth, WA, 6000, Australia; Centre for Clinical Research in Emergency Medicine, Harry Perkins Institute of Medical Research, Perth, WA, 6000, Australia; School of Population and Global Health, University of Western Australia, Perth, 6009, Australia; Queensland Alliance for Environmental Health Sciences (QAEHS), The University of Queensland, Dutton Park, Brisbane, Queensland, 4102, Australia; National Centre for Youth Substance Use Research, The University of Queensland, St Lucia, 4072, Australia

## Abstract

The growing emergence of new psychoactive substances (NPS) challenges traditional approaches to drug monitoring, highlighting a need for innovative surveillance strategies. We explored the potential to better understand the NPS market in Australia by combining wastewater analysis with toxicosurveillance programs. Focussing on a time of year of likely higher NPS and drug use, influent wastewater samples were collected from four Australian states (South Australia, Victoria, Queensland, and New South Wales) over the New Year period from 2019–2020 to 2024–2025. Clinical samples presenting between 15 December and 15 January each period from the toxicosurveillance systems Emerging Drugs Network of Australia (including Western Australia, South Australia, and Queensland), Emerging Drugs Network of Australia Victoria (Victoria) and Prescription, Recreational and Illicit Substance Evaluation—PRISE (New South Wales) were extracted. In total, 294 wastewater and 117 clinical samples were included in this work. A total of 46 individual NPS were found in both datasets. This included: 21 only in wastewater, 36 only in clinical, and 12 in both. Overall, 743 instances of NPS were found in all wastewater samples and 171 in clinical data. In wastewater samples, N, N-dimethylpentylone (54%), mitragynine (52%), and bromazolam (45%) were the most frequently detected. In the clinical samples, the most common were bromazolam (44%) and clonazolam (21%). Overall, synthetic cathinones and plant-based compounds were more prevalent in wastewater, and synthetic opioids and benzodiazepines in clinical samples. Differences in detected NPS classes illustrate the complementary nature of these surveillance systems. Higher wastewater concentrations may indicate broader use and lower acute harm than drugs identified clinically. Integrating these datasets with additional sources such as surveys, coronial records, police seizures, and drug-checking services can strengthen NPS monitoring.

## Introduction

Since the mid-2000s, a large and diverse range of synthetic substances, often designed as legal alternatives to more established illicit drugs, have entered the global drug supply. For pragmatic reasons these substances have been termed new psychoactive substances (NPS). Their advent has challenged traditional approaches to drug monitoring such as population-based or targeted surveys with people who use drugs because exposure is often unintentional, and the content of these substances is often unknown to people who use drugs.

In 2024, the United Nations Office on Drugs and Crime (UNODC) recorded 755 individual NPS, which is the highest count of individual NPS per year ever recorded [1]. Synthetic Cathinones comprised the largest NPS group reported (33%), followed by synthetic cannabinoid receptor agonists (24%), hallucinogens (14%), and synthetic opioids (9%). In Australia, multiple data sources highlight an upward trend in the availability, use, and associated harms of NPS [2–5]. This includes growing concern about the emergence of potent synthetic opioids (such as nitazenes) and novel benzodiazepines [6].

Effective public health and harm reduction responses rely on objective surveillance methods with high capability to identify emerging NPS of potential public health significance. Recent studies have shown the utility of such surveillance programs in Australia. For example, a retrospective study of all deaths due to drug toxicity in Australia using the National Coronial Information System identified the contribution of novel benzodiazepines in drug toxicity deaths [7]. A total of 258 cases were found between 2013 and 2025, with 225 occurring since 2020, primarily involving etizolam (125) and bromazolam (98) [8]. A similar study conducted in Victoria, Australia extracted data from the Victorian Overdose Deaths Register to examine the novel benzodiazepines that contributed to Victorian overdose deaths between 2009 and 2023 [9]. Among the 140 reported deaths, etizolam (60) and bromazolam (39) were found to be the most frequent benzodiazepines contributing to overdoses [9].

The importance of toxicosurveillance strategies has become increasingly apparent in recent years, particularly in response to emerging concerns surrounding nitazenes and novel benzodiazepines. For example, a cluster of multi-drug intoxications involving xylazine, nitazenes, and novel benzodiazepines was reported in South Australia [10]; 20 unintentional acute opioid poisonings associated with nitazene exposure were reported in New South Wales between 2021 and 2025 [11]; and one of the earliest reported cases of nitazene intoxication in Australia occurred in Victoria in 2021 [12]. These state-specific toxicosurveillance systems, except the PRISE program and other toxicosurveillance programs centrally coordinated by the NSW Ministry of Health, are now part of the Emerging Drugs Network of Australia (EDNA), which recently reported 32 nitazene-related presentations nationally between 2020 and 2024 [13]. Such systems have become integral in harm reduction strategies, exemplified by the issuance of a public Drug Alert after the Emerging Drugs Network of Australia—Victoria (EDNAV) identified five distinct novel benzodiazepines in a cluster of six adults presenting to hospital following the use of counterfeit benzodiazepines [14]. Wastewater analysis is an additional, complementary data source that provides an objective, population-level approach to assessing community drug consumption. It has been used to assess national drug consumption in Australia since 2016 [15] and recently shown to detect NPS internationally, including nitazenes [16–18]. In this article, we describe complementary insights from wastewater analysis and clinical toxicosurveillance systems to identify NPS across five Australian states between 2019 and 2025.

## Methods

### Sample collection

Wastewater and clinical data were collected from regional and capital city sites from five Australian states over the New Year period (December–January from 2019/20 to 2024/25) (Supplementary Table 1). This particular time period was selected because previous studies demonstrate an increase in illicit substance consumption related to holidays and music festivals [19, 20].

Wastewater samples were collected annually for 7–10 days over six New Year surveillance periods from 17 metropolitan and regional sites in Queensland, South Australia, New South Wales, and Victoria. In total, there were 294 individual wastewater samples (36, 32, 44, 72, 69, and 41 over each yearly sampling campaign; Table 2).

**Table 1:** Inclusion criteria and sample matrix for each toxicosurveillance system.

	EDNA	EDNAV	PRISE
Inclusion criteria	Patients with severe *and/or* unusual clinical features of reported or suspected illicit drug toxicity *and/or* patients presenting as part of a suspected cluster of recreational drug poisonings	Suspected opioid exposure, severe toxicity, and cases where there was a discrepancy between reported exposure and observed clinical effects	Patients with severe *and/or* unusual clinical features of reported or suspected illicit drug toxicity *and/or* patients presenting as part of a suspected cluster of recreational drug poisonings
Age criteria	16 years and over in WA and Qld; 18yrs and over in SA	16 years and older	No age criteria
Samples collected for analysis	Blood	Blood	Blood and urine (if available)

**Table 2:** Number of samples included in this study.

Year	Count									
	WA^a^		SA		Vic		NSW		Qld	
	WW	C	WW	C	WW	C	WW	C	WW	C
2019–2020			36							
2020–2021		2	32			11				
2021–2022		3	44			6				
2022–2023		7	44	3		23	21	1	7	2
2023–2024		3	28	2	7	28	21	1	13	1
2024–2025		3			13	17	7	3	21	
Total		18	184	5	20	85	49	5	41	3

a C, Clinical; NSW, New South Wales; Qld, Queensland; SA, South Australia; Vic, Victoria; WA, Western Australia; WW, Wastewater.

EDNA and EDNAV are multi-site surveillance programs monitoring illicit drug-related emergency department presentations across Australia and Victoria, respectively [21, 22]. They involve comprehensive blood testing for a wide spectrum of pharmaceutical, traditional illicit and NPS compounds and metabolites, alongside the collection of relevant clinical information.

Prescription, Recreational, and Illicit Substance Evaluation (PRISE) is a clinical and public health program that provides access to extensive toxicology testing to NSW acute care services for cases of severe and unusual substance related toxicity or clusters of overdoses [23]. Notifications to the program come from a range of sources that include clinical toxicology services, the NSW Poisons Information Centre and alcohol and other drug treatment services. Additional intelligence inputs which prompt active case finding include syndromic surveillance of unplanned presentations to emergency departments, coronial toxicology results, and police seizures.

Clinical data from individual cases where at least one NPS was present in blood and/or urine samples were included from EDNA, EDNAV, and PRISE. These covered hospital sites in Western Australia, South Australia, Queensland (EDNA), Victoria (EDNAV), and New South Wales (PRISE) (Table 1). Further information on ethics, participants and procedures have been previously described [11, 21, 24–26].

### Clinical case selection

Each toxicosurveillance system has their own specific criteria for case inclusion (Table 1). To ensure comparability between the wastewater and toxicosurveillance datasets, clinical data were extracted with dates of presentation between 15 December and 15 January. In total, 116 individual cases were included in this study (Table 2).

### Sample analysis

Wastewater samples were processed and analyzed using validated liquid chromatography–mass spectroscopy methods, previously described [18, 27–29] and specified in the supporting information, together with method validation parameters (Supplementary Table 2).

The clinical samples were submitted to state-specific laboratories and tested for NPS, with details explained elsewhere [2]. Detection, identification, and quantification methods for the clinical samples are based on each laboratory’s internal protocols and, where available, certified reference materials. Briefly, to ensure consistency in screening and detection across different states, a *Primary Drug Target List* has been established, containing around 200 compounds that all laboratories are capable of detecting. Analytical instrumentation utilized across states includes liquid chromatography–quadrupole time of flight mass spectrometry, liquid chromatography–tandem mass spectrometry and gas chromatography–triple quadrupole mass spectrometry.

The analytical target lists used for both wastewater and clinical testing were periodically reviewed and updated throughout the study period to reflect changes in the NPS market, the availability of reference standards, and emerging intelligence on substances of concern.

To avoid double-counting, metabolites that were co-detected with the parent compound in the same sample were not included. For example, pentylone when with N, N-dimethylpentylone, alpha-hydroxyetizolam when with etizolam, and 8-aminoclonazolam when with clonazolam. However, there were some complex situations in which some NPS could be manufacturing by-products. For example, phenazepam and desalkylgidazepam may be used as synthetic precursors of clobromazolam and bromazolam, respectively [2]. In the clinical cases of this study, desalykylgidazepam and phenazepam were only detected together with clobromazolam and bromazolam. However, it is impossible to differentiate with certainty between a synthetic by-product and an NPS so each has been included as individual NPS.

### Statistical analysis

Analysis and visualization of all data was conducted using GraphPad Prism 10. All categorical variables were presented as frequencies and proportions. Any NPS were organized into the following classes based upon NPS substance groups from the United Nations Office of Drugs and Crime (UNODC).

### Role of funding sources

Funders of the study had no role in design, collection, analysis or interpretation of this manuscript.

## Results

A total of 46 individual NPS were found across both datasets: 21 in wastewater, 36 in clinical, and 12 in both (Table 3). Overall, 743 instances of NPS were found in all wastewater samples and 171 in clinical data. The yearly breakdowns are included in the supporting information (Supplementary Tables 3 and 4). Up to 24 NPS in the clinical dataset and 11 in the wastewater samples detected were within a single time period. Conversely, there were several compounds that were found across multiple years that included bromazolam, clonazolam, flualprazolam, N, N-dimethylpentylone, and mitragynine.

**Figure 1:**
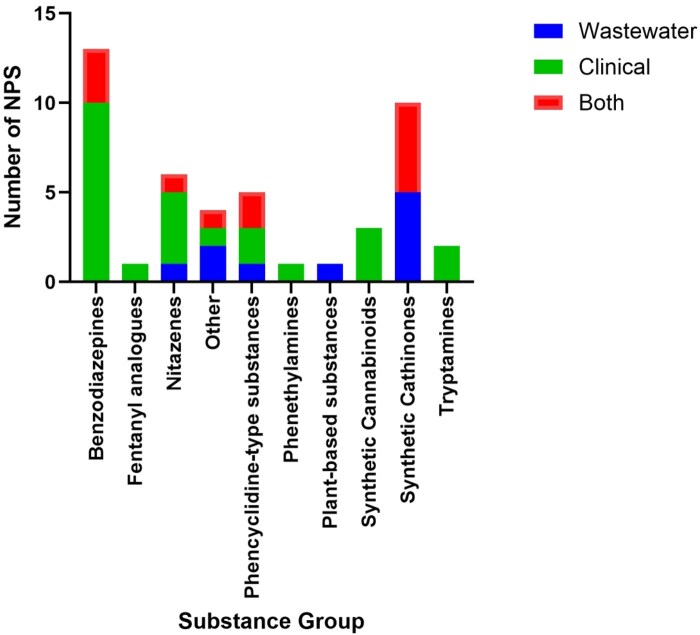
Counts of each NPS substance group found across clinical and wastewater datasets.

A total of 10 NPS substance groups were found across both datasets. Benzodiazepines (*n* = 13) and synthetic cathinones (*n* = 10) were the most commonly detected substance groups of NPS (Fig. 1). There were five groups that were not detected in both datasets. Plant-based compounds (mitragynine) were only found in wastewater samples, while tryptamines (dimethyltryptamine and 4-HO-MET), phenethylamines (PMMA) and synthetic cannabinoids (5C-APINACA, ADB-BUTINACA and Cumyl-PeGACLONE) were only found in clinical data. In terms of individual NPS, bromazolam (*n* = 52; 45% detection frequency), clonazolam (*n* = 24; 21%) and desalkylgidazepam (*n* = 14; 12%) were most often detected in clinical cases, while N-N-dimethylpentylone (*n* = 160; 54% detection frequency), mitragynine (*n* = 154; 52%), and bromazolam (*n* = 131; 45%) were most common in the wastewater samples.

## Discussion

Monitoring NPS poses ongoing challenges for public health surveillance, particularly in contexts of rapid market change and heterogeneous patterns of use. While wastewater and clinical data may appear to be independent datasets capturing different aspects of the NPS consumption spectrum, their partial overlap and divergence provide complementary insights into both prevalence and harm.

### Complementary datasets

Some compounds that were frequently detected in both wastewater and clinical data (Supplementary Fig. 1), with bromazolam the clearest example. Several synthetic cathinones (e.g. N, N-dimethylpentylone, methylone, and eutylone) as well as etizolam, protonitazene, 2F-2-oxo-PCE and xylazine were also detected in both. In general, synthetic cathinones and plant-based compounds (e.g. mitragynine) were more likely to be found in wastewater, while opioids, synthetic cannabinoids, and benzodiazepines were commonly seen in clinical cases.

Differences in detection of NPS are likely due to program design differences. Compounds such as synthetic cathinones and mitragynine have lower clinical toxicity and therefore are less likely to require medical attention, despite potentially widespread use detectable in wastewater. However, it is important to note that the metabolite 7-hydroxymitragynine can be considerably more potent than mitragynine and may present a higher risk profile [30]. In contrast, synthetic opioids, particularly nitazenes, are incredibly potent (up to 500 times more potent than morphine and 20 times more potent than fentanyl [31]), while severity of exposure is one of the key determinants for the purposive sampling of EDNA, EDNAV, and PRISE. These differences in detected compounds and NPS substance groups highlight the complementary strengths of these two surveillance systems: wastewater analysis may reflect broader community use, including substances less likely to cause severe morbidity, while clinical data capture drugs associated with acute harm. The ability of wastewater analysis to cover a catchment of more than 1 million inhabitants means that it could be used to estimate a denominator in calculating rates of serious adverse events presenting to hospitals. For example, if a nitazene is detected in one of the toxicosurveillance programs but not in wastewater, it is likely that use is isolated and thus not indicative of widespread community harm. Alternatively, if a nitazene is found in wastewater but not in clinical cases, it could provide early warning of community use [16].

Together, wastewater analysis and clinical data can act as two slices of a “Swiss cheese” model for an early warning system of NPS use in Australia. This model illustrates how several imperfect and biased models of data collection can complement each other to provide a coordinated response to emerging threats and prevent harm. Similar models have been adopted in healthcare [32] and gained prominence during the COVID-19 pandemic, where the importance of multiple interventions such as social distancing, mask wearing, and vaccinations could work together to reduce transmission risk [33]. It has recently been suggested to tackle antimicrobial resistance [34]. Further strengthening this model will require the integration of additional complementary data sources such as surveys, coronial and police seizure data, needle and syringe residue analysis, drug-checking services, and consumer-reported information, co-designed in partnership with people who use drugs to support a more comprehensive early-warning system.

### Advantages and limitations

Both datasets offer complementary strengths as surveillance tools for monitoring the new psychoactive substance (NPS) market in Australia. Wastewater-based analysis functions effectively as a pooled community urine sample, providing a non-invasive, anonymized, and relatively unbiased snapshot of drug consumption at the population level. In the current work it must be noted that wastewater treatment plant catchments do not represent entire states, and not all states were sampled in each year, limiting generalizability to broader drug markets. With sampling conducted over the New Year period, increased transient populations associated with holiday destinations and music festivals may have contributed to wastewater inputs, potentially biasing detections toward certain substance classes. Additionally, COVID-19-related public health restrictions were in place during portions of the 2020/21 and 2021/22 study periods in some jurisdictions, which may have altered social activities, event attendance, and patterns of NPS consumption, potentially influencing both wastewater detections and clinical presentations.

Compared with other large-scale surveillance approaches such as self-report surveys, wastewater monitoring is cost-effective with short turnaround times between sample analysis that allows near-real time (days–weeks) monitoring of emerging trends. With sustained longitudinal sampling, wastewater analysis can also be used to assess temporal trends in consumption and evaluate the impact of public health or regulatory interventions [18, 35]. Importantly, wastewater captures both harmful and non-harmful patterns of use, including recreational, occasional, and non-problematic consumption, and may reflect drug use among individuals who do not engage with surveys or present to healthcare services.

In contrast, clinical toxicosurveillance data provide objective, individual-level evidence of NPS associated with acute intoxication and overdose requiring emergency or intensive care. These data directly characterize substances linked to adverse health outcomes and, in some cases, may represent the earliest signal of emerging NPS that cause harm, even when community-level prevalence remains low. Established collaborations between hospitals and forensic laboratories at local and interstate levels further enhance the rapid dissemination of intelligence to frontline clinicians and public health authorities, supporting timely harm-minimization responses to emerging drug-related threats [22]. However, methodological and sampling considerations should be taken into account when interpreting these findings. Differences in study design and analytical scope limit the ability to make direct, absolute comparisons between datasets. For example, drug-related hospital presentations are typically only screened for NPS when exposure is suspected, meaning EDNA, EDNAV, and PRISE data are not representative of all drug-related hospitalizations or the broader population of people who use illicit drugs. In addition, EDNA and EDNAV analyze blood samples only, whereas PRISE analyses both blood and urine when available, potentially extending the detection window for some substances. Furthermore, given the rapidly evolving nature of the NPS market, the analytical target lists used for both wastewater and clinical testing were periodically updated throughout the study period to incorporate newly identified substances. As a result, temporal differences in detection frequency may partly reflect changes in analytical coverage in addition to changes in substance prevalence (Table 3).

**Table 3:** Frequency of individual NPS detected across both datasets.

Compound	Class	Wastewater (*n* = number of samples with positive detection (detection frequency, %))	Clinical Data (*n* = number of cases with positive detection (detection frequency, %))
2F-2-oxo-PCE	Phencyclidine-type substances	41 (14)	5 (4)
2F-Deschloroketamine	Phencyclidine-type substances	21 (7)	
2-Methyl-4'-(methylthio)-2-Morpholinopropiophenone^a^	Other		1 (1)
3,4-Methylenedioxy PV8	Synthetic Cathinones	44 (15)	
3-Hydroxy-PCP^a^	Phencyclidine-type substances		2 (2)
3-Methylmethcathinone	Synthetic Cathinones	10 (3)	
4-OH-MET^a^	Tryptamines		1 (1)
5C-APINACA^a^	Synthetic Cannabinoids		1 (1)
Acetyl fentanyl	Fentanyl analogues		2 (2)
ADB-BUTINACA^a^	Synthetic Cannabinoids		1 (1)
Bromazolam	Benzodiazepines	131 (45)	52 (44)
Clobromazolam	Benzodiazepines		9 (8)
Clonazolam	Benzodiazepines	3 (1)	24 (21)
Cumyl-PeGACLONE	Synthetic Cannabinoids		1 (1)
Delorazepam^a^	Benzodiazepines		1 (1)
Desalkylgidazepam	Benzodiazepines		14 (12)
Deschloroetizolam	Benzodiazepines		6 (5)
Deschloro-N-Ethyl ketamine	Phencyclidine-type substances		1 (1)
Dibutylone	Synthetic Cathinones	72 (24)	
Diclazepam^a^	Benzodiazepines		1 (1)
Dimethyltryptamine^a^	Tryptamines		1 (1)
Ethylene etonitazene	Nitazenes		1 (1)
Ethylone	Synthetic Cathinones	21 (7)	
Etizolam	Benzodiazepines	5 (2)	8 (7)
Etodesnitazene	Nitazenes		2 (2)
Etonitazepyne	Nitazenes	5 (2)	
Eutylone	Synthetic Cathinones	42 (14)	1 (1)
Flualprazolam	Benzodiazepines		6 (5)
Flubromazepam	Benzodiazepines		4 (3)
Flunitrazolam	Benzodiazepines		1 (1)
Mephedrone	Synthetic Cathinones	13 (4)	
Methcathinone	Synthetic Cathinones	^b^	1 (1)
Methiopropamine	Other	2 (1)	
Methoxetamine	Phencyclidine-type substances	2 (1)	1 (1)
Methylone	Synthetic Cathinones	10 (3)	5 (4)
Metonitazene	Nitazenes		1 (1)
Mitragynine	Plant-based substances	154 (52)	
N, N-Dimethylpentylone	Synthetic Cathinones	160 (54)	9 (8)
N-Ethylpentylone	Synthetic Cathinones	2 (1)	1 (1)
Nitrazolam^a^	Benzodiazepines		1 (1)
N-Pyrrolidino etonitazene	Nitazenes		1 (1)
Phenazepam^a^	Benzodiazepines		2 (2)
Phenibut	Other	7 (2)	
PMMA	Phenethylamines		1 (1)
Protonitazene	Nitazenes	6 (2)	1 (1)
Xylazine	Other	8 (3)	1 (1)
Total		743	172

a At the time of collection, these substances were not included in methods of analyzing wastewater.

b Methcathinone was found in all wastewater samples. However, it is likely to have been formed in sewer from (pseudo)ephedrine [36, 37].

## Conclusion

This study demonstrates the value of integrating wastewater-based surveillance with clinical toxicosurveillance to characterize the evolving NPS landscape in Australia. A total of 46 NPS were found, with 21 in wastewater, 36 in clinical data, and 12 in both. While there were some differences between the datasets, with synthetic opioids appearing more often in the clinical findings and synthetic cathinones and plant-based NPS in the wastewater, the benzodiazepine bromazolam was consistently found in both, suggesting both community exposure and acute toxicity. By capturing both population-level patterns of drug exposure and individual-level presentations associated with acute harm, these complementary approaches provide a more nuanced understanding of emerging substances than either method alone.

## Supplementary Material

bkag071_Supplementary_Data

## Data Availability

The data underlying this article will be shared on reasonable request to the corresponding author.

## Acknolwedgements

The authors sincerely thank the wastewater treatment plant operators for their help in collecting samples and the support provided by the lab staff who performed the extraction of the samples. The authors also thank SCIEX for the use of their instrument. EDNA and EDNAV thank all project site investigators and the analytical services provided by ChemCentre, Forensic Science SA, Forensic Toxicology, Forensic Pathology and Coronial Services, Queensland Health and the Victorian Institute of Forensic Medicine. Shaun Greene is also acknowledged for his help in collating the data. The data presented relies on the contributions of those with lived and living experience of drug use, and PRISE would like to acknowledge and thank the community for this contribution. PRISE would also like to acknowledge the analytical services provided by Forensic & Analytical Science Service, NSW Health Pathology.
