# Supplementary material for: Complementary insights from wastewater surveillance and the toxicology findings of clinical presentations involving new psychoactive substances in Australia
Source: J Anal Toxicol. 2026 Aug 5;50(7):bkag071. doi: 10.1093/jat/bkag071 (PMC13481691; doi:10.1093/jat/bkag071)
Supplement: Supplementary file 1 [file bkag071_supplementary_data.pdf]

Supporting Information

Complementary insights from wastewater surveillance and the toxicology findings of clinical presentations involving new psychoactive substances in Australia

Table S1: Number of sites involved in this study. M = metropolitan/capital city; R: regional

Year	WA		SA		Vic		NSW		Qld	
	WW	C	WW	C	WW	C	WW	C#	WW	C
2019-20		3 M	4 M							
2020-21		4 M, 1R	4 M			12M, 4 R				
2021-22		4 M, 1R	4 M	4 M		13M, 4 R	3 M		1 R	
2022-23		4 M, 1R	4 M	4 M		13M, 4 R	3 M		2 R	1 M
2023-24		4 M, 1R	4 M	4 M	1 (M)	13M, 4 R	3 M		1M, 1R	1 M
2024-25		4 M, 1R		4 M	2 (R)	14M, 4R	1 R		3 M	1 M

#The PRISE program is available to the whole of NSW, therefore collection sites are not limited to certain hospitals. Clinical data was included if the location of the case presentation was within the three metropolitan wastewater catchment areas sampled. For 2024-25 an additional regional wastewater site was sampled, any cases occurring in this catchment area were included in the clinical data for 2024-25. (R: regional, M: metropolitan, WW: Wastewater, C: Clinical cases)

Wastewater Sample preparation (adapted from (Laimou-Geraniou et al., 2025))

Influent wastewater samples (100 mL) were filtered under vacuum using glass microfibre filter paper (GF/A 1.6 µm, Whatman, Kent, U.K.). Before extraction, samples were spiked with deuterated internal standards (illicit drug reference standard mixture, spiked at 150 ng/L), and their pH was adjusted to 4.5–5 using hydro- chloric acid (37 %). Subsequently, solid phase extraction (SPE) cartridges (UCT XtracT DAU, 500 mg/6 mL; UCT Inc., Bristol, PA, USA) were conditioned with methanol (6 mL) and sodium acetate buffer (20 mM, 6 mL). The samples were then loaded on the cartridges under gravity, and the cartridges were successively washed with sodium acetate buffer (20 mM, 6 mL), 0.1 M acetic acid (2 mL), and methanol (6 mL). The sorbent was dried under vacuum for 15 min. A mixture of dichloromethane/isopropanol/aqueous ammonia (80:16:4 v/v/v, 6 mL) was used to elute the analytes of interest from the cartridges. The eluent was then evaporated to approximately 200 µL under nitrogen at 40 ◦C, after which 1 % HCl in methanol (20 µL) was added, and the sample evaporated to dryness. The dry residue was reconstituted with 0.1 % formic acid in methanol (20 µL) and 0.1 % formic acid in ultrapure water (80 µL), giving a final volume of 100 µL (i.e., a 1000 times concentration factor).

Instrumental Analysis

Quantitative analysis was performed using a liquid chromatography-tandem mass spectrometry (LC-MS/MS) method utilising a Shimadzu Nexera LC-40 (Kyoto, Japan) coupled to a SCIEX Triple Quad 7500 system (Framingham, MA, USA). Chromatographic separation was achieved using a Phenomenex Kinetex Biphenyl column (50 mm x 2.1 mm, 2.6 µm) fitted with a Phenomenex security guard ultra cartridges HPLC Biphenyl 2.1 mm ID columns at a flow rate of 0.45 mL/min and an injection volume of 2 µL. Mobile phase A consisted of 95 % ultrapure water with 5 % MeOH and 0.1 % formic acid, while mobile phase B consisted of 95 % MeOH with 5 % ultrapure water and 0.1 % formic acid. The chromatographic gradient was as follows: 5 % B at 0 min and held for 1 min, linear increase to 100 % B at 7 min and held for 2 min, then decreased to 5 % B at 9.1 min and kept steady for the final 1.9 min to equilibrate the system. The total runtime was 11 min. The mass spectrometer was run in scheduled multiple reaction monitoring (sMRM) mode in positive ionisation mode, with a 30 s retention time window around the expected retention time of each analyte.

Quantification and Method Validation (adapted from (Laimou-Geraniou et al., 2025; Nadarajan et al., 2025)

To confirm a compound in the wastewater samples, both transitions had to be present, with an ion ratio within 30 % and a retention time within 0.1 min of the reference standard, following SANTE guidelines. As analyte-specific internal standards were unavailable for all analytes, the concentration of NPS compounds were determined based on the peak area ratios between native and surrogate internal standards compared to an external calibration curve. For the analytes detected in this method, MDMA-d5 was utilised as a surrogate to enable semi-quantification.

Method validation included selectivity, recovery, matrix effect, limits of detection (LOD), and limits of quantification (LOQ) in wastewater (Table S1). The linearity and R-squared values were assessed using a 10 % MeOH solution. Both LOD and LOQ were evaluated by analysing duplicate wastewater samples that had been spiked at in-sample concentrations of 1, 5, 10 and

50 ng/L. The LOD was determined as the lowest level in which both analyte transitions could be seen with a signal-to-noise ratio of 3, with LOQ at a signal-to-noise ratio of 10. To provide a more accurate estimation, where the signal-to-noise level at the lowest level was above 3 (or 10), the LOD (or LOQ) was back- calculated to determine the level at which a signal-to-noise ratio of 3 (or 10) could be achieved. Extraction recovery was evaluated in pre- and post-extracted samples, as previously described (Matuszewski et al., 2003). The matrix effect was assessed by comparing the post-extracts and the neat solvent. Extraction recovery and matrix effect were both determined in triplicates.

Quality control for the quantitative method includes control at 100 ng/L and 250 ng/L for every 10 samples. As part of quality control assurance, a double-blank injection without internal standards was injected every five samples. All standards were freshly made on the day of analysis.

Table S2: Figures of merit for all NPS detected in wastewater in the current study. Data compiled from previous publications (Bade et al., 2020, 2019; Nadarajan et al., 2025)

NPS	LOD (ng/L)	LOQ (ng/L)	Reportable range (ng/L)	R-square	% Recovery at 10 ng/L (Mean ± SD)			% Matrix effect (10 ng/L)
2-fluorodeschloroketamine	0.83	2.5	2.5 – 1000	0.9957	102.4	±	8.7	39
2-oxo-PCE	0.26	0.77	0.77 – 1000	0.9985	97.2	±	7.7	40
3,4-Methylenedioxy PV8	0.11	0.33	0.33 – 1000	0.9984	114.2	±	1.9	41
3-Methylmethcathinone	0.85	2.5	2.5 – 500	0.9960	103.2	±	9.0	41
Bromazolam	0.20	0.62	0.62 – 1000	0.9963	121.7	±	7.3	126
Clonazolam	2.5	7.5	7.5 – 1000	0.9949	31.7	±	4.5	145
Dibutylone	0.27	0.81	0.81 – 1000	0.9994	112.1	±	5.9	53
Ethylone	0.02	0.2	0.1 – 21.5	0.9969	96	±	4	81
Etizolam	2.1	6.5	6.5 – 1000	0.9961	95.8	±	5.6	58
Etonitazepyne	0.81	2.4	2.4 – 500	0.9936	105.5	±	2.9	43
Eutylone	0.21	0.63	0.63 – 500	0.9976	101.5	±	5.7	140
Mephedrone	0.38	1.1	1.1 – 1000	0.9980	83	±	10	103
Methcathinone	0.44	1.3	1.3 - 500	0.9990	106.1	±	9.6	81
Methiopropamine	0.01	0.1	0.01 - 40	0.9981	102	±	8	111
Methoxetamine	0.01	0.05	0.01 - 40	0.9990	86	±	10	96
Methylone	0.3	1	0.1 – 21.25	0.9989	109	±	9	90
Mitragynine	3.8	11	11 – 1000	0.9919	106.2	±	8.1	-
N,N-dimethylpentylone	0.37	1.1	1.1 – 1000	0.9988	112.2	±	5.2	136
N-ethylpentylone	0.75	2.2	2.2 - 1000	0.9983	109.6	±	3.2	17
Pentylone	0.20	0.61	0.61 – 1000	0.9997	110.8	±	6.5	56
Phenibut	1.0	3.2	3.2 – 500	0.9940	41	±	12	151
Protonitazene	0.27	0.82	0.82 – 500	0.9978	110	±	16	49

Table S3: All NPS detected from clinical data from 2020-21 to 2024-25

Compound	Class	2020-21	2021-22	2022-23	2023-24	2024-25	Total
	Number of cases (n)	13	9	36	36	23	117
2F-2-oxo-PCE	Phencyclidine-type substances				5		5
2F-deschloroketamine	Phencyclidine-type substances						
2-Methyl-4'-(methylthio)-2-Morpholinopropiophenone	Other			1			1
3,4-Methylenedioxy PV8	Synthetic Cathinones						
3-hydroxy-PCP	Phencyclidine-type substances	2					2
4-OH-MET	Indolalkylamines					1	1
5C-APINACA	Synthetic Cannabinoids	1					1
Acetyl fentanyl	Fentanyl analogues				1	1	2
ADB-BUTINACA	Synthetic Cannabinoids					1	1
Bromazolam	Benzodiazepines		1	14	26	11	52
Clobromazolam	Benzodiazepines			7	1		8
Clonazolam	Benzodiazepines	2	5	12	3	2	24
Cumyl-PeGACLONE	Synthetic Cannabinoids	1					1
Delorazepam	Benzodiazepines	1					1
Desalkylgidazepam	Benzodiazepines				9	5	14
Deschloroetizolam	Benzodiazepines			3	2	1	6
Deschloro-N-Ethyl ketamine	Phencyclidine-type substances					1	1
Dibutylone	Synthetic Cathinones						
Diclazepam	Benzodiazepines	1					1
Dimethyltryptamine	Indolalkylamines	1					1
Ethylene etonitazene	Nitazenes					1	1

Ethylone	Synthetic Cathinones						
Etizolam	Benzodiazepines	4			3	1	8
Etodesnitazene	Nitazenes		1	1			2
Etonitazepyne	Nitazenes						
Eutylone	Synthetic Cathinones	1					1
Flualprazolam	Benzodiazepines	2	1		1	2	6
Flubromazepam	Benzodiazepines			2	1	1	4
Flunitrazolam	Benzodiazepines				1		1
Mephedrone	Synthetic Cathinones						
Methcathinone	Synthetic Cathinones	1					1
Methiopropamine	Other						
Methoxetamine	Phencyclidine-type substances	1					1
Methylone	Synthetic Cathinones				2	3	5
Metonitazene	Nitazenes				1		1
Mitragynine	Plant-based						
N,N-Dimethylpentylone	Synthetic Cathinones			6	3		9
N-ethylpentylone	Synthetic Cathinones	1					1
Nitrazolam	Benzodiazepines				1		1
N-pyrrolidino etonitazene	Nitazenes				1		1
Phenazepam	Benzodiazepines			1	1		2
PMMA	Phenethylamines				1		1
Protonitazene	Nitazenes				1		1
Xylazine	Other				1		1
TOTAL		19	8	48	66	31	172

Table S4: All NPS detected from wastewater data from 2019-20 to 2024-25

Compound	Class	2019-20	2020-21	2021-22	2022-23	2023-24	2024-25	Total
	Number of samples (n)	36	32	44	72	69	41	294
2F-2-oxo-PCE	Phencyclidine-type substances						41	41
2F-deschloroketamine	Phencyclidine-type substances				12	1		21
2-Methyl-4'-(methylthio)-2-Morpholinopropiophenone	Other							
3,4-Methylenedioxy PV8	Synthetic Cathinones				44			44
3-hydroxy-PCP	Phencyclidine-type substances							
3-Methylmethcathinone	Synthetic Cathinones				10			10
4-OH-MET	Indolalkylamines							
5C-APINACA	Synthetic Cannabinoids							
Acetyl fentanyl	Fentanyl analogues							
ADB-BUTINACA	Synthetic Cannabinoids							
Bromazolam	Benzodiazepines				27	63	41	131
Clobromazolam	Benzodiazepines							
Clonazolam	Benzodiazepines			3				3
Cumyl-PeGACLONE	Synthetic Cannabinoids							
Delorazepam	Benzodiazepines							
Desalkylgidazepam	Benzodiazepines							
Deschloroetizolam	Benzodiazepines							
Deschloro-N-Ethyl ketamine	Phencyclidine-type substances							
Dibutylone	Synthetic Cathinones				44	28		72
Diclazepam	Benzodiazepines							
Dimethyltryptamine	Indolalkylamines							

Ethylene etonitazene	Nitazenes							
Ethylone	Synthetic Cathinones	18	3					21
Ethylphenidate	Phenidates							
Etizolam	Benzodiazepines			2	3			5
Etodesnitazene	Nitazenes							
Etonitazepyne	Nitazenes					5		5
Eutylone	Synthetic Cathinones	8	29	5				42
Flualprazolam	Benzodiazepines							
Flubromazepam	Benzodiazepines							
Flunitrazolam	Benzodiazepines							
Mephedrone	Synthetic Cathinones		5		8			13
Methcathinone	Synthetic Cathinones							
Methiopropamine	Other		2					2
Methoxetamine	Phencyclidine-type substances		2					2
Methylone	Synthetic Cathinones		10					10
Metonitazene	Nitazenes							
Mitragynine	Plant-based			44	26	43	41	154
N,N-Dimethylpentylone	Synthetic Cathinones				70	49	41	160
N-ethylpentylone	Synthetic Cathinones		2					2
Nitrazolam	Benzodiazepines							
N-pyrrolidino etonitazene	Nitazenes							
Phenazepam	Benzodiazepines							
Phenibut	Other				7			7
PMMA	Phenethylamines							
Protonitazene	Nitazenes					6		6
Xylazine	Other						8	8
TOTAL		26	53	46	251	195	172	743

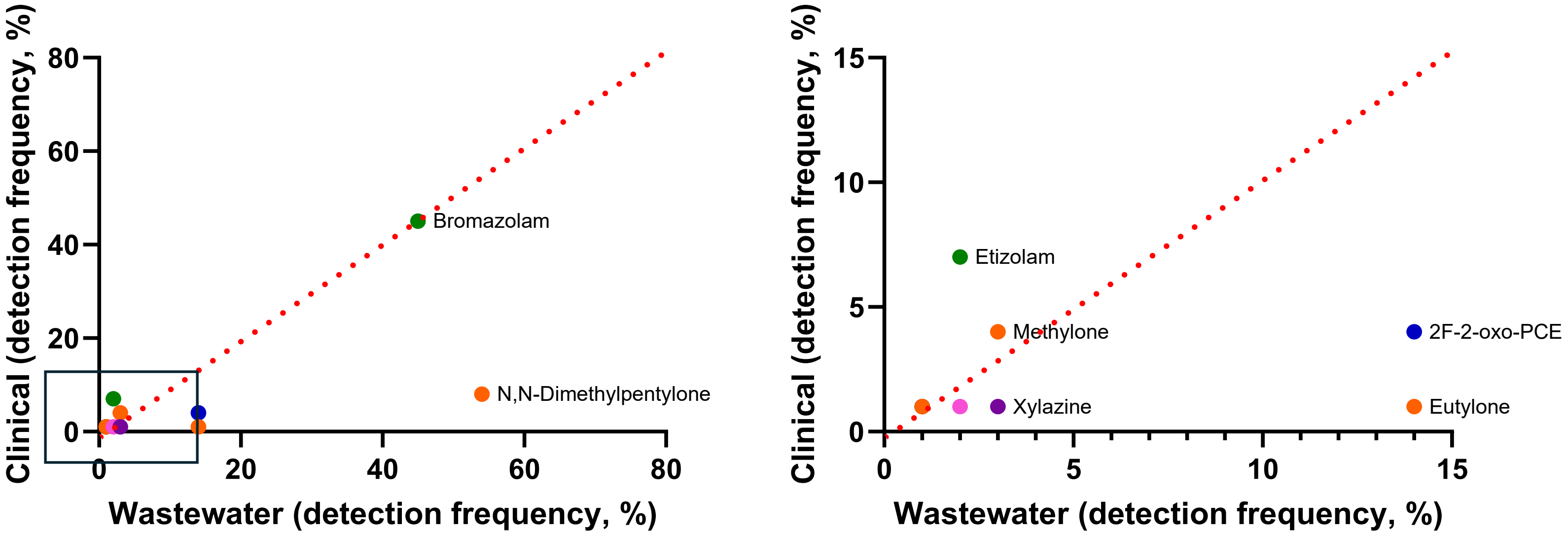

Figure S1: Detection frequency correlation between clinical data (y axis) and wastewater (x axis) for all compounds that were found in both data sets. Red line has a slope of 1, indicating perfect correlation. Compounds found above the line were more commonly found in clinical samples and those below were common in wastewater. Most highly detected compounds are labelled, with colours reflecting NPS class (green : benzodiazepines; orange: synthetic cathinones; blue: phencyclidine-type substances, pink: synthetic opioids and purple: other). The right-hand figure zooms in on the less detected compounds (less than 15% detected).

References

Bade, R., Abdelaziz, A., Nguyen, L., Pandopulos, A.J., White, J.M., Gerber, C., 2020. Determination of 21 synthetic cathinones, phenethylamines, amphetamines and opioids in influent wastewater using liquid chromatography coupled to tandem mass spectrometry. Talanta 208, 120479. https://doi.org/10.1016/j.talanta.2019.120479

Bade, R., Ghetia, M., Nguyen, L., Tscharke, B.J., White, J.M., Gerber, C., 2019. Simultaneous determination of 24 opioids, stimulants and new psychoactive substances in wastewater. MethodsX 6, 953–960. https://doi.org/10.1016/j.mex.2019.04.016

Laimou-Geraniou, M., Nadarajan, D., Bade, R., Heath, E., 2025. Spatiotemporal trends of new psychoactive substances in Slovenia through influent wastewater analysis. J. Hazard. Mater. Adv. 19, 100781. https://doi.org/10.1016/j.hazadv.2025.100781

Matuszewski, B.K., Constanzer, M.L., Chavez-Eng, C.M., 2003. Strategies for the assessment of matrix effect in quantitative bioanalytical methods based on HPLC-MS/MS. Anal. Chem. 75, 3019–3030. https://doi.org/10.1021/ac020361s

Nadarajan, D., Laimou-Geraniou, M., Chappell, A., Baduel, C., Bijlsma, L., Boogaerts, T., Burgard, D.A., Castiglioni, S., Ceolotto, N., Driver, E.M., Sodre, F.F., Kassinos, D.F., Quintana, H.F., Gerber, C., Gracia-Lor, E., Gracia-Marín, E., Halden, R.U., Heath, E., Huchthausen, J., Kasprzyk-Hordern, B., Keller, E.L., Lai, F.Y., Löve, A.S.-C., Matias, J., Ocenaskova, V., Oh, J.-E., Oluseyi, T., Phung, K., Pineda-Castro, M., Psichoudaki, M., Salgueiro-Gonzalez, N., Silvino-Gomes, C., Nascimento, F.M.S., Subedi, B., Thomaidis, N., Van Wichelen, N., Wang, D., Yargeau, V., Cresswell, S., Mueller, J.F., Bade, R., 2025. Wastewater analysis as a global toxicovigilance tool for the monitoring of new psychoactive substances. Water Res. 124847. https://doi.org/10.1016/j.watres.2025.124847
